# A morphological and phylogenetic analysis of dematiaceous hyphomycete strains of *Distoseptispora* (*Distoseptisporaceae*, *Distoseptisporales*) and *Kirschsteiniothelia* (*Kirschsteiniotheliaceae*, *Pleosporales*) in southern China

**DOI:** 10.3897/mycokeys.133.194698

**Published:** 2026-05-27

**Authors:** Yongze He, Xingsheng Wang, Xigang Yan, Wenwen Liu, Yang Jiang, Congcong Ai, Jinyan Sheng, Xiuguo Zhang, Shi Wang

**Affiliations:** 1 College of Life Sciences, Shandong Normal University, Jinan, 250358, China College of Life Sciences, Shandong Normal University Jinan China; 2 Shandong Provincial Key Laboratory for Biology of Vegetable Diseases and Insect Pests, College of Plant Protection, Shandong Agricultural University, Taian, 271018, China College of Plant Protection, Shandong Agricultural University Taian China

**Keywords:** *
Dothideomycetes
*, morphology, new species, *
Sordariomycetes
*, taxonomy

## Abstract

Dematiaceous hyphomycetes are widely distributed throughout the world. They can live either saprophytically or parasitically and are commonly found growing on dead wood, in soil, and in aquatic environments. During an ongoing survey of saprophytic fungi in multiple southern provinces of China, two *Distoseptispora*-like and one *Kirschsteiniothelia*-like strains were isolated from decaying wood. Five barcodes (i.e., ITS, SSU, LSU, *RPB2*, and *TEF1*) were amplified and sequenced. Of these markers, ITS, LSU, *RPB2*, and *TEF1* were selected for *Distoseptispora*, whereas ITS, LSU, and SSU were used for *Kirschsteiniothelia*. Based on maximum likelihood (ML) and Bayesian inference (BI) phylogenetic analyses, combined with morphological characteristics, three new species, *Distoseptispora linchunlingensis*, *D. xinganensis*, and *Kirschsteiniothelia guiyangensis*, are proposed. This study provides detailed illustrations, phylogenetic trees, and morphological descriptions to clarify the taxonomic status of the three new species, thereby improving our understanding of the diversity of dematiaceous hyphomycetes in Hainan, Guangxi, and Guizhou provinces, China.

## Introduction

Dematiaceous hyphomycetes constitute a widely distributed and ecologically distinct fungal group, characterized by dark, melanin-rich hyphae and spores that enhance stress tolerance and environmental persistence (Rai et al. 2021). Commonly found on decaying wood and in soil, they serve as key saprotrophs and endophytes, playing pivotal roles in litter decomposition, nutrient mineralization, and promoting plant resilience in forest soil ecosystems (Xia et al. 2014). Despite their broad occurrence and functional significance, most species remain poorly sampled. Many are recalcitrant to cultivation using conventional methods, leaving their diversity, distribution, and functional traits in forest habitats incompletely documented (Jones et al. 2014; Hyde et al. 2016). Although current research primarily employs high-throughput sequencing to reveal these uncharacterized lineages, formal taxonomic description and functional characterization of dematiaceous hyphomycetes still lag significantly behind their actual diversity (Shams-Ghahfarokhi et al. 2014).

Soil and plant debris serve as critical microhabitats for diverse fungal communities, in which saprotrophic fungi drive the decomposition of lignocellulosic materials and mediate carbon cycling (Guarro et al. 2012; Taylor and Sinsabaugh 2015). In particular, fungi associated with plant substrates such as dead branches and leaf litter have garnered increasing attention owing to their ecological importance and promising biotechnological applications (Yeasmin et al. 2015). During surveys of fungal diversity in Hainan, Guizhou, and Guangxi provinces of China, three regions characterized by rich subtropical biodiversity, numerous fungal strains were isolated from soil and decaying plant tissues. Among these isolates, three novel species were identified using a combination of morphological examination and molecular phylogenetic analysis.

The genus Distoseptispora (Sordariomycetes, Xylariales) was first established by Su et al. (2016). It is morphologically defined by hyaline to pale brown, macronematous and mononematous conidiophores, integrated cylindrical to clavate conidiogenous cells, and characteristic distoseptate conidia. The conidia are acrogenous, solitary or catenate, hyaline to pigmented, and distinguished by false septa (Chomnunti et al. 2014; Sun et al. 2021). Tibpromma et al. isolated and identified two new species, *D. thailandica* and *D. xishuangbannaensis*, from the decaying leaves of *Pandanus* hosts and revised the phylogenetic tree of the family *Distoseptisporaceae* (Tibpromma et al. 2018). Meanwhile, Monkai et al. described *D. hydei*, a species isolated from decaying bamboo culms in Phitsanulok, Thailand, which is characterized by distinctive conidial features: 7–9-distoseptate conidia are formed in the apical region, ovoid to fusiform in shape, and surrounded by a hyaline gelatinous sheath (Monkai et al. 2020). Currently, 124 entries linked to *Distoseptispora* are listed in Index Fungorum (http://www.indexfungorum.org/, accessed on 17 February 2026). Members of this genus are widely distributed across terrestrial ecosystems, occurring predominantly as saprotrophs on decaying plant material. Several species have been reported from China, Thailand, and other tropical and subtropical regions (Sun et al. 2020; Wang et al. 2022).

In contrast, Kirschsteiniothelia (Dothideomycetes, Kirschsteiniotheliales) was established by Hawksworth (1985) with *K. aethiops* as the type species (Hawksworth 1985). This genus is morphologically distinguished by epigenous to semi-immersed, dark brown to black ascomata, bitunicate asci, and brown to dark brown, septate ascospores (Boonmee et al. 2014; Wijayawardene et al. 2018). Currently, 79 entries linked to *Kirschsteiniothelia* are recorded in Index Fungorum (http://www.indexfungorum.org/, accessed on 17 February 2026). The taxonomic position of *Kirschsteiniothelia* has undergone several revisions. The genus was initially assigned to *Pleosporaceae* (Hawksworth 1985; Barr 1987), later transferred to *Pleomassariaceae* (Barr, 1992), and then accommodated in the newly established family *Kirschsteiniotheliaceae* by Boonmee et al. (2014). Subsequently, Hernández-Restrepo et al. (2017) established the monotypic order *Kirschsteiniotheliales* due to its distant phylogenetic placement relative to other orders within *Dothideomycetes* (Hernandez-Restrepo et al. 2017).

Multi-locus phylogenetic analysis has become a standard approach in fungal systematics, enabling accurate species delimitation and phylogenetic placement by integrating molecular data with morphological characteristics (Maharachchikumbura et al. 2015; Hyde et al. 2016). In this study, three new fungal species were isolated and identified from decaying plant samples collected in Hainan, Guangxi, and Guizhou provinces, China: *D. linchunlingensis* from Hainan, *D. xinganensis* from Guangxi, and *K. guiyangensis* from Guizhou. These new taxa are delimited based on morphological distinctions and phylogenetic analyses of combined ITS, LSU, *RPB2*, and *TEF1* sequence datasets. This study formally describes and illustrates these three new species and discusses their phylogenetic relationships within their respective genera.

## Materials and methods

### Sample collection and treatment

Decaying wood samples were collected from Hainan, Guangxi, and Guizhou provinces, China. Detailed information, including collector, collection date, locality, altitude, latitude, and longitude, was recorded for each sample. The collected decaying wood was cut into suitable segments and placed in 13 × 13 cm square Petri dishes lined with filter paper. Sterile water was added, and the samples were incubated at room temperature for 2–3 weeks. Samples were observed every 3 days, and additional sterile water was supplemented to maintain appropriate humidity. Conidia were isolated from decaying wood using a sterile inoculation needle and cultured on potato dextrose agar (PDA) medium (14 g agar, 20 g glucose, and 200 g potato infusion per liter of distilled water; pH 7.0). After colony development, single colonies were picked with a sterilized inoculating loop and subcultured onto fresh PDA medium to obtain pure strains.

### Morphological and cultural characterization

When the colonies had grown to the 7^th^, 14^th^, 21^st^, and 28^th^ day, a digital camera (Canon Powershot G7X; Beijing, China) was used to photograph both the front and back of the culture medium. A stereo microscope (Olympus SZX10; Beijing, China) was used to observe the overall colony morphology and examine sporulation. Spore-producing colonies were selected and prepared as temporary slide mounts, and the microscopic morphology of the fungus was observed using a microscope (Olympus BX53). Next, a high-definition digital camera (Olympus DP80) was used to capture images of microscopic structures, such as conidiophores, conidiogenous cells, and conidia. All strains were stored in 10% glycerol (v/v) in sterile tubes at 4 °C. Voucher specimens are kept in the Herbarium Mycologicum Academiae Sinicae at the Institute of Microbiology, Chinese Academy of Sciences, Beijing, China (HMAS). In addition, cultures derived from the holotype have been preserved in the Shandong Agricultural University Culture Collection (SAUCC). The morphological description and taxonomic information of the new species have been uploaded to Fungal Names (https://nmdc.cn/fungalnames/, accessed on 15 October 2025).

### DNA extraction, PCR amplification, and sequencing

Fungal DNA was extracted using cetyl trimethyl ammonium bromide (CTAB) as the main method (Wang et al. 2023; Zhang et al. 2025). Once the mycelium reached the appropriate length, 0.2 g was harvested using a scalpel and transferred to a 1.5 mL microcentrifuge tube. Preheated CTAB buffer (800 μL) was added to the tube and inverted several times to mix well. The sample was ground in a grinder for 5 min each time, three times in total. The centrifuge tube was placed in a 65 °C water bath for 2 h and then centrifuged at 12,000 rpm for 10 min. A pipette was used to transfer 700 μL of the supernatant into a new 1.5 mL centrifuge tube. Chloroform:isoamyl alcohol (700 μL; 24:1) was added in a fume hood and then placed on a shaker for 20 min. After centrifugation at 12,000 rpm for 10 min, 400 μL of the supernatant was transferred to a new 1.5 mL centrifuge tube. Isopropanol (400 μL) was added, mixed thoroughly, and incubated at room temperature for 20 min. The sample was centrifuged for 40 min, the supernatant was discarded, and the DNA pellet was collected (Guo et al. 2000). The extracted fungal DNA was amplified by PCR using primer sets targeting the ITS, LSU, SSU, *RPB2*, and *TEF1* regions. The primer sets used were ITS: ITS5/ITS4; SSU: NS1/NS4; LSU: LR0R/LR5; *RPB2*: fRPB2-5F/fRPB2-7cR; and *TEF1*: EF1-983F/EF1-2218R.

PCR amplifications were carried out in a 25 μL reaction volume containing 2 μL of DNA template, 1 μL each of forward and reverse primers, 9.5 μL of double-distilled water (ddH_2_O) and 2.5 μL of 2× Taq Plus Master Mix (with dye; Yeasen Biotechnology, Shanghai, China; Cat. No. 10154ES03). PCR products were separated on a 2% agarose gel stained with ethidium bromide and visualized under UV light to confirm amplification (Ma et al. 2022). PCR primer synthesis and DNA sequencing were performed by Sangon Biotech Co., Ltd. (Shanghai, China). Multiple sequence alignment of the obtained sequencing data was carried out using MAFFT 7 software, and the aligned sequences were further adjusted and verified using MEGA7 to ensure alignment accuracy (Kumar et al. 2016) (Table 1). The multi-gene sequences of the three new species have been uploaded to NCBI GenBank, with the accession numbers to be provided upon request. The GenBank accessions referenced in this study were collated and summarized in Tables 2, 3.

**Table 1.** PCR primers, sequences, and reaction conditions used in this study.

Loci	PCR primers	Sequence (5'–3')	PCR cycles
ITS	ITS4	GGA AGT AAA AGT CGT AAC AAG G	(95 °C at 30 s, 55 °C at 30 s, 72 °C at 1 min) × 35 cycles
	ITS5	TCC TCC GCT TAT TGA TAT GC	
SSU	NS1	GTA GTC ATA TGC TTG TCT C	(95 °C at 30 s, 52 °C at 60 s, 72 °C at 90 s) × 35 cycles
	NS4	CTT CCG TCA ATT CCT TTA AG	
LSU	LR0R	GTA CCC GCT GAA CTT AAG C	(95 °C at 30 s, 55 °C at 50 s, 72 °C at 1 min) × 35 cycles
	LR5	TCC TGA GGG AAA CTT CG	
*RPB2*	f*RPB2*-5F	CAT CGA GAA GTT CGA GAA GG	(95 °C at 45 s, 57 °C at 50 s, 72 °C at 90 s) × 40 cycles
	f*RPB2*-7cR	GGA RGT ACC AGT SAT CAT GTT	
*TEF1*	EF1-983F	GCY CCY GGH CAY CGT GAY TTY AT	(94 °C at 30 s, 55 °C at 50 s, 72 °C at 1 min) × 35 cycles
	EF1-2218R	AT GAC ACC RAC RGC RAC RGT YTG	

**Table 2.** GenBank accession numbers used in the phylogenetic analysis of *Distoseptispora*.

Taxon	Strain	GenBank accession numbers			
		ITS	LSU	RPB2	TEF1
*Aquapteridospora fusiformis*	MFLUCC 18–1606*	MK828652	MK849798	N/A	MN194056
*A. lignicola*	MFLUCC 15–0377*	MZ868774	KU221018	MZ892986	MZ892980
*Distoseptispora adscendens*	HKUCC 10820	N/A	DQ408561	DQ435092	N/A
*D. amniculi*	MFLUCC 17–2129*	MZ868770	MZ868761	MZ892982	N/A
*D. appendiculata*	MFLUCC 18–0259*	MN163009	MN163023	N/A	MN174866
*D. aqualignicola*	KUNCC 21–10729*	OK341186	ON400845	OP413474	OP413480
*D. aquamyces*	KUNCC 21–10732*	OK341187	OK341199	OP413476	OP413482
*D. aquatica*	MFLUCC 18–0646	MK828648	MK849793	N/A	MN194052
*D. aquisubtropica*	GZCC 22–0075*	ON527933	ON527941	ON533685	ON533677
*D. arecacearum*	MFLUCC 23–0212	OR354399	OR510860	OR481048	OR481045
*D. atroviridis*	GZCC 20–0511*	MZ868772	MZ868763	MZ892984	MZ892978
*D. atroviridis*	GZCC 19–0531	MW133915	MZ227223	N/A	MZ206155
*D. bambusae*	MFLUCC 20–0091*	MT232713	MT232718	MT232881	MT232880
*D. bambusicola*	GZCC 21–0667*	MZ474873	MZ474872	N/A	OM272845
*D. bangkokensis*	MFLUCC 18–0262*	MZ518205	MZ518206	N/A	OK067246
*D. bawanglingensis*	SAUCC WZS13-1*	PQ799295	PQ804721	PQ849357	PQ849363
*D. bawanglingensis*	SAUCC WZS13-2	PQ799296	PQ804722	PQ849358	PQ849364
*D. cangshanensis*	MFLUCC 16–0970*	MG979754	MG979761	N/A	MG988419
*D. caricis*	CPC 36498*	MN562124	MN567632	MN556805	N/A
*D. changjiangensis*	SAUCC WZS14-1*	PQ799297	PQ804723	PQ849359	PQ849366
*D. changjiangensis*	SAUCC WZS14-1	PQ799298	PQ804724	PQ849360	PQ849365
*D. chinensis*	GZCC 21–0665*	MZ474871	MZ474867	N/A	MZ501609
*D. chishuiensis*	GZCC 23- 0729*	PP584670	PP584767	N/A	PP663310
*D. clematidis*	MFLUCC 17–2145*	MT310661	MT214617	MT394721	N/A
*D. clematidis*	HJAUP C1319	PQ211102	PQ211110	PQ303676	PQ303681
*D. combreticola*	CGMCC 3.27721*	PQ189782	PQ184737	N/A	N/A
*D. combreticola*	UESTCC 23.0470	PQ191050	PQ184725	N/A	N/A
*D. crassispora*	KUMCC 21–10726*	OK310698	OK341196	OP413473	OP413479
*D. curvularia*	KUMCC 21–10725*	OK310697	OK341195	OP413472	OP413478
*D. cylindricospora*	DLUCC 1906*	OK491122	OK513523	N/A	OK524220
*D. daanyuanensis*	SAUCC 12326-1*	PV670056	PV670405	N/A	PV708057
*D. daanyuanensis*	SAUCC 12326-2	PV670057	PV670406	N/A	PV708058
*D. davidalangii*	UESTCC 24.0236*	PQ189781	PQ184736	PQ380000	PQ346519
*D. davidalangii*	UESTCC 23.0473	PQ191048	PQ184723	PQ379953	PQ346499
*D. davidii*	HKAS 145866*	PV820360	PV856177	N/A	N/A
*D. dehongensis*	KUMCC 18–0090*	MK085061	MK079662	N/A	MK087659
*D. dinghuensis*	ZHKUCC 23-0958*	PQ037957	PQ037956	PQ035180	PQ035181
*D. dipterocarpi*	MFLUCC22–0104*	OP600053	OP600052	OP595140	N/A
*D. dujuanhuensis*	KUNCC 23–13772*	PQ845849	PV536297	PX233767	PX238245
*D. effusa*	GZCC 19–0532*	MW133916	MZ227224	N/A	MZ206156
*D. eleiodoxae*	MFLUCC 23–0214	OR354398	OR510859	OR481047	OR481044
*D. euseptata*	MFLUCC 20–0154*	MW081539	MW081544	MW151860	N/A
*D. euseptata*	DLUCC S2024	MW081540	MW081545	MW084996	MW084994
*D. fasciculata*	KUMCC 19–0081*	MW286501	MW287775	N/A	MW396656
*D. fluminicola*	DLUCC 0391	MG979755	MG979762	N/A	MG988420
*D. fluminicola*	DLUCC 0999	MG979756	MG979763	N/A	MG988421
*D. fujianensis*	HJAUP C2509*	PQ211095	PQ211103	PQ303679	PQ303682
*D. fujianensis*	HJAUP C2513	PQ211098	PQ211106	PQ303680	PQ303683
*D. fusiformis*	GZCC 20–0512*	MZ868773	MZ868764	MZ892985	MZ892979
*D. ganzhouensis*	HJAUP C1090*	PQ211100	PQ211108	N/A	PQ303687
*D. gasaensis*	HJAUP C2034*	OQ942896	OQ942891	N/A	OQ944455
*D. guanshanensis*	HJAUP C1063*	OQ942894	OQ942898	OQ944458	OQ944452
*D. guizhouensis*	GZCC 21–0666*	MZ474868	MZ474869	MZ501611	MZ501610
*D. guttulata*	MFLU 17–0852*	MF077543	MF077554	N/A	MF135651
*D. hainanensis*	GZCC 22 2047*	OR427328	OR438894	OR449119	OR449122
*D. heptapleuricola*	CGMCC 3.27740*	PQ189783	PQ184738	PQ380001	PQ346520
*D. heptapleuricola*	UESTCC 23.0474	PQ191051	PQ184726	PQ379954	PQ346500
*D. hongheensis*	KUNCC 23-14299*	PQ845851	PV536299	PX233769	PX238247
*D. hyalina*	MFLUCC 17–2128*	MZ868769	MZ868760	MZ892981	MZ892976
*D. hydei*	MFLUCC 20-0481*	MT734661	MT742830	N/A	N/A
*D. jianfenglingensis*	SAUCC WZS65-3*	PQ799299	PQ804725	PQ849361	PQ849367
*D. jianfenglingensis*	SAUCC WZS65-4	PQ799300	PQ804726	PQ849362	PQ849368
*D. jingdongensis*	KUNCC 23-13382*	PQ845852	PX245753	PX233770	PX238248
*D. jingdongensis*	KUNCC 23-14297	PQ845853	PV536301	PX233771	PX238249
*D. jinghongensis*	HJAUP C2120*	OQ942897	OQ942893	N/A	OQ944456
*D. keviniana*	HKAS:145874*	PV820358	N/A	N/A	N/A
*D. keviniligustrina*	CGMCC 3.27722*	PQ191052	PQ184727	PQ379955	PQ346501
*D. lancangjiangensis*	DLUCC 1864*	MW723055	MW879522	MW882260	N/A
*D. lanceolatispora*	GZCC 22-2045*	OR427329	OR438895	OR449120	OR449123
*D. leonensis*	HKUCC 10822	N/A	DQ408566	DQ435089	N/A
*D. licualae*	MFLUCC 14–1163A*	ON650686	ON650675	N/A	ON734007
*D. licualae*	MFLUCC 14–1163B*	ON650687	ON650676	N/A	ON734008
***D. linchunlingensis***	SAUCC1738201*	**PZ100104**	**PZ112124**	**PZ121388**	**PZ121394**
***D. linchunlingensis***	**SAUCC1738202**	**PZ100105**	**PZ112125**	**PZ121389**	**PZ121395**
*D. lignicola*	GZCC 19-0529	MW133911	MZ227219	N/A	MZ206152
*D. lignicola*	HFJAU 0705*	MK828651	MK849797	N/A	N/A
*D. liupanshuiensis*	GZCC 23-0730*	PP584669	PP584766	N/A	PP663309
*D. longispora*	HFJAU 0705*	MH555359	MH555357	N/A	N/A
*D. longissima*	GMBC5339*	PV932968	PV932987	PX373356	PX392330
*D. longissima*	GMBC5340	PV932969	PV932988	PX373357	PX392331
*D. longnanensis*	HJAUP C1040*	OQ942887	OQ942886	N/A	OQ944451
*D. martinii*	CGMCC 3.18651*	KU999975	KX033566	N/A	N/A
*D. meilingensis*	JAUCC 4727*	OK562390	OK562396	N/A	OK562408
*D. meilingensis*	JAUCC 4728*	OK562391	OK562397	N/A	OK562409
*D. menghaiensis*	HJAUP C2045*	OQ942890	OQ942900	N/A	N/A
*D. menghaiensis*	HJAUP C2170	OQ942899	OQ942888	OQ944461	OQ944457
*D. mengsongensis*	HJAUP C2126*	OP787876	OP787874	N/A	OP961937
*D. monospora*	HKAS 145690*	PQ898696	PQ898699	PV001672	PV001669
*D. muchuanensis*	CGMCC 3.27444	PQ067919	PQ067750	N/A	PQ278571
*D. multiseptata*	MFLUCC 15–0609*	KX710145	KX710140	N/A	MF135659
*D. multiseptata*	MFLU 17–0856	MF077544	MF077555	MF135644	MF135652
*D. nabanheensis*	HJAUP C2003*	OP787873	OP787877	N/A	OP961935
*D. nanchangensis*	HJAUP C1074*	OQ942889	OQ942895	OQ944460	OQ944454
*D. nanpingensis*	HJAUP C2517*	PQ211096	PQ211104	PQ303678	N/A
*D. narathiwatensis*	MFLUCC 23–0216	OR354400	OR510861	OR481049	OR481046
*D. neorostrata*	MFLUCC 18–0376*	MN163008	MN163017	N/A	N/A
*D. nonrostrata*	KUNCC 21–10730*	OK310699	OK341198	OP413475	OP413481
*D. obclavata*	MFLUCC 18–0329*	MN163012	MN163010	N/A	N/A
*D. obpyriformis*	MFLUCC 17–1694*	N/A	MG979764	MG988415	MG988422
*D. obpyriformis*	DLUCC 0867	MG979757	MG979765	MG988416	MG988423
*D. olivaceoviridis*	MFLU 24-0290	PQ568144	PQ569325	N/A	N/A
*D. pachyconidia*	KUMCC 21–10724*	OK310696	OK341194	OP413471	OP413477
*D. palmarum*	MFLUCC 18–1446*	MK085062	MK079663	MK087670	MK087660
*D. phangngaensis*	MFLUCC 16–0857*	MF077545	MF077556	N/A	MF135653
*D. phragmiticola*	GUCC 220201*	OP749887	OP749880	OP752699	OP749891
*D. phragmiticola*	GUCC 220202*	OP749888	OP749881	OP752700	OP749892
*D. pseudoaquisubtropica*	HKAS 136251*	PV820361	PV856178	N/A	N/A
*D. pulchra*	KUNCC 23 16269	PQ427202	PQ431179	PX233820	PX238335
*D. quinqueseptata*	GMBC5341*	PV932966	PV932985	PX373358	PX392328
*D. quinqueseptata*	GMBC5342	PV932967	PV932986	PX373359	PX392329
*D. rayongensis*	MFLUCC 18–0415*	MH457172	MH457137	MH463255	MH463253
*D. rayongensis*	MFLUCC 18–0417	MH457173	MH457138	MH463256	MH463254
*D. rostrata*	MFLUCC 16–0969*	MG979758	MG979766	MG988417	MG988424
*D. rostrata*	DLUCC 0885	MG979759	MG979767	N/A	MG988425
*D. saprophytica*	MFLUCC 18–1238*	MW286506	MW287780	MW504069	MW396651
*D. septata*	GZCC 22–0078*	ON527939	ON527947	ON533690	ON533683
*D. sichuanensis*	KUNCC 23-15519*	PP584672	PP584769	N/A	PP663312
*D. sinensis*	HJAUP C2044*	OP787878	OP787875	N/A	OP961936
*D. solitaria*	HKAS 145916*	PV820362	PV856179	N/A	N/A
*D. songkhlaensis*	MFLUCC 18–1234*	MW286482	MW287755	N/A	MW396642
*D. suae*	CGMCC 3.24262*	OQ874968	OQ732679	OQ870341	OR367670
*D. submersa*	MFLUCC 16-0946*	MG979760	MG979768	MG988418	MG988426
*D. subtropica*	HJAUP C2528*	PQ211099	PQ211107	PQ303677	PQ303684
*D. subtropica*	HJAUP C2535	PQ211097	PQ211105	N/A	PQ303685
*D. suoluoensis*	MFLUCC 17–0224*	MF077546	MF077557	N/A	MF135654
*D. suoluoensis*	MFLUCC 17–0854	MF077547	MF077558	MZ945510	N/A
*D. tectonae*	MFLUCC 12–0291*	KX751711	KX751713	KX751708	KX751710
*D. tectonae*	MFLU 20–0262	MT232714	MT232719	N/A	N/A
*D. tectonigena*	MFLUCC 12–0292*	KX751712	KX751714	KX751709	N/A
*D. terrestris*	HJAUP M2539*	PV448667	PV450538	N/A	PV469764
*D. thailandica*	MFLUCC 16–0270*	MH275060	MH260292	N/A	MH412767
*D. thysanolaenae*	KUN–HKAS 112710	MW723057	MW879524	N/A	MW729783
*D. thysanolaenae*	KUN–HKAS 102247*	MK045851	MK064091	N/A	MK086031
*D. tongrensis*	GMBC5343*	PV932970	PV932989	PX373360	N/A
*D. tongrensis*	GMBC5344	PV932971	PV932990	PX373361	N/A
*D. tropica*	GZCC 22–0076*	ON527935	ON527943	ON533687	ON533679
*D. uncariicola*	UESTCC 24.0229	PQ189784	PQ184739	PQ380002	PQ346521
*D. uncariicola*	UESTCC 23.0420	PQ191056	PQ184731	PQ379957	PQ346503
*D. vaginae*	GZAAS 25-0541*	PV820363	PV856180	N/A	N/A
*D. velvetica*	GZAAS 25-0535*	PV820364	PV856181	N/A	N/A
*D. verrucosa*	GZCC 20–0434*	MZ868771	MZ868762	MZ892983	MZ892977
*D. wuyishanensis*	HJAUP C2515*	PV448666	PV450537	PV469759	PV469763
*D. wuzhishanensis*	GZCC 22–0077*	ON527938	ON527946	N/A	ON533682
*D. xinpingensis*	KUNCC 22–12667*	OQ874970	OQ732681	OQ870340	OR367671
***D. xinganensis***	SAUCC1835501*	**PZ100106**	**PZ112126**	**PZ121390**	**PZ121396**
***D. xinganensis***	**SAUCC1835502**	**PZ100107**	**PZ112127**	**PZ121391**	**PZ121397**
*D. xishuangbannaensis*	KUMCC 17–0290*	MH275061	MH260293	MH412754	MH412768
*D. yichunensis*	HJAUP C1065*	OQ942885	OQ942892	OQ944459	OQ944453
*D. yongxiuensis*	JAUCC 4725*	OK562388	OK562394	N/A	OK562406
*D. yunjushanensis*	JAUCC 4724*	OK562392	OK562398	N/A	OK562410
*D. yunjushanensis*	HJAUP C1307	PQ211101	PQ211109	PQ303675	PQ303686
*D. yunnanensis*	MFLUCC20–0153*	MW081541	MW081546	MW151861	MW084995
*D. zhejiangensis*	HJAUP C2588*	PV448668	PV450539	N/A	PV469765
*D. zunyiensis*	GZAAS 23-0810*	PQ248941	N/A	PQ614176	PQ605053

Notes: New species established in this study are shown in bold. Ex-type strains are indicated using “*.” N/A: not available.

**Table 3.** GenBank accession numbers used in the phylogenetic analysis of *Kirschsteiniothelia*.

Taxon	Strain	GenBank accession numbers		
		ITS	LSU	RPB2
*Pseudorobillarda eucalypti*	MFLUCC 12-0422	KF827451	KF827457	KF827463
*P. phragmitis*	CBS 398.61	MH858101	MH869670	EU754104
*Kirschsteiniothelia acutisporum*	MFLU 21-0127*	OP120780	ON980758	ON980754
*K. agumbensis*	NFCCI 5714*	PP029048	N/A	PP029049
*K. aquatica*	MFLUCC 16-1685*	MH182587	MH182594	MH182618
*K. arasbaranica*	IRAN 2509C	KX621986	KX621987	KX621988
*K. arasbaranica*	IRAN 2508C*	KX621983	KX621984	KX621985
*K. atra*	DEN	MG602687	N/A	N/A
*K. atra*	CBS 109.53	N/A	AY016361	AY016344
*K. atra*	MFLUCC 16-1104	MH182583	MH182589	MH182615
*K. atra*	S-783	MH182586	MH182595	MH182617
*K. atra*	MFLUCC 15-0424	KU500571	KU500578	KU500585
*K. atra*	GZCC 23-0731	PQ248940	PQ248936	PQ248932
*K. bulbosapicalis*	GZCC 23-0732*	PQ248937	PQ248933	PQ248929
*K. cangshanensis*	MFLUCC 16-1350	MH182584	MH182592	N/A
*K. chiangmaiensis*	MFLU 23-0358*	OR575473	OR575474	OR575475
*K. crustacea*	MFLU 21-0129*	MW851849	MW851854	N/A
*K. dendryphioides*	KUNCC 10431*	OP626354	PQ248935	PQ248931
*K. dendryphioides*	KUNCC 10499	PQ248938	N/A	N/A
*K. dujuanhuensis*	KUNCC 22-12671*	OQ874971	OQ732682	OQ875039
*K. dushanensis*	GZCC 19-0415*	OP377845	MW133830	MW134610
*K. ebriosa*	CBS H-23379*	N/A	LT985885	N/A
*K. ebriosa*	CBS 143842	N/A	LT985884	N/A
*K. emarceis*	MFLU 10-0037*	HQ441570	HQ441571	N/A
*K. esperanzae*	T.Raymundo 6581*	OQ877253	OQ880482	N/A
*K. extensa*	MFLU 21-0130*	MW851850	MW851855	N/A
*K. fluminicola*	MFLUCC 16-1263*	MH182582	MH182588	N/A
*K. ganzhouensis*	HJAUP C1209*	PP505546	PP506568	PP527763
*K. ganzhouensis*	HJAUP C1210	PQ456024	PQ443751	PQ443763
*K. ganzhouensis*	HJAUP C1211	PQ456025	PQ443752	PQ443764
*K. guangdongensis*	ZHKUCC 22-0233*	OR164946	OR164974	N/A
***K. guiyangensis***	**SAUCCGY3703**	PZ100112	PZ100114	PZ100118
***K. guiyangensis***	SAUCCGY3704*	PZ100113	PZ100115	PZ100119
*K. guizhouensis*	GZCC 24-0034*	PQ404852	PQ404856	PQ404859
*K. guizhouensis*	GZCC 24-0041	PQ404853	N/A	PQ404860
*K. hydei*	HKAS 145701	PQ819629	PQ819638	PX210251
*K. inthanonensis*	MFLUCC 23-0277*	OR762773	OR762781	OR764784
*K. inthanonensis*	HJAUP C1502	PQ456029	PQ443756	PQ443768
*K. inthanonensis*	HJAUP C1503	PQ456030	PQ443757	PQ443769
*K. jiangxiensis*	HJAUP C1273*	PP505548	PP506566	PP506565
*K. jiangxiensis*	HJAUP C1274	PQ456026	PQ443753	PQ443765
*K. jiangxiensis*	HJAUP C1275	PQ456027	PQ443754	PQ443766
*K. jiulianshanensis*	HJAUP C1313*	PP505549	PP506562	PP506563
*K. jiulianshanensis*	HJAUP C1314	PQ456028	PQ443755	PQ443767
*K. laojunensis*	KUN L 88727*	PP081651	PP081658	N/A
*K. lignicola*	MFLUCC 10-0036*	HQ441567	HQ441568	HQ441569
*K. linzhiensis*	HKAS 144539*	PV484695	PQ675402	PQ675362
*K. linzhiensis*	HKAS 144540	PV484696	PQ675403	PQ675363
*K. longirostrata*	GZCC 23-0733*	PQ248939	PQ248934	PQ248930
*K. longisporum*	UESTCC 24.0190*	PQ038266	PQ038273	PQ046108
*K. mucosa*	KUNCC 24-17953*	PQ152660	PQ152608	PQ218174
*K. mucosa*	KUNCC 24-18127	PV647851	PQ152609	PQ218310
*K. nabanheensis*	HJAUP C2006	OQ023274	OQ023275	OQ023037
*K. nabanheensis*	HJAUP C2004*	OQ023197	OQ023273	OQ023038
*K. phoenicis*	MFLU 18-0153	NR_158532	NG_064508	N/A
*K. phoenicis*	MFLUCC 18-0216*	MG859978	MG860484	MG859979
*K. pini*	UESTCC24.0131*	PP835321	PP835315	PP835318
*K. puerensis*	ZHKUCC 22-0272	OP450978	OP451018	OP451021
*K. puerensis*	ZHKUCC 22-0271*	OP450977	OP451017	OP451020
*K. ramus*	GZCC 23-0596*	NR_190260	NG_243331	N/A
*K. rostrata*	MFLUCC 15-0619	KY697280	KY697276	KY697278
*K. rostrata*	MFLU 15-1154*	NR_156318	NG_059790	NG_063633
*K. rostrata*	MFLUCC 16-1124	N/A	MH182590	N/A
*K. saprophytica*	MFLUCC 23-0275*	OR762774	OR762783	N/A
*K. saprophytica*	MFLUCC 23-0276	OR762775	OR762782	N/A
*K. septemseptatum*	MFLU 21-0126*	OP120779	ON980757	ON980752
*K. sichuanensis*	UESTCC 24.0127*	PP785368	PP784322	N/A
*K. longiconidiophora*	KUNCC 23-13756*	OR589303	OR600952	OR743201
*K. longiconidiophora*	KUNCC 23-14559	OR589302	OR600951	N/A
*Kirschsteiniothelia*. sp.	KUNCC 23-13755*	OR589301	OR600949	OR743199
*Kirschsteiniothelia*. sp.	UTHSCSA DI22-44	ON191447	ON191450	N/A
*Kirschsteiniothelia*. sp.	UTHSCSA DI22-45	ON191448	ON191449	N/A
*Kirschsteiniothelia*. sp.	7020611638	**MZ380314**	**MZ380317**	**N/A**
*Kirschsteiniothelia*. sp.	E38	**MN912317**	**MN912273**	**N/A**
*Kirschsteiniothelia*. sp.	CSN604	MT813881	N/A	N/A
*Kirschsteiniothelia*. sp.	CSN602	MT813880	N/A	N/A
*K. spatiosum*	MFLU 21-0128*	N/A	N/A	ON980753
*K. submersa*	S-481	N/A	MH182591	MH182616
*K. submersa*	S-601	MH182585	MH182593	N/A
*K. submersa*	MFLUCC 15-0427*	KU500570	KU500577	KU500584
*K. tectonae*	MFLUCC 12-0050*	KU144916	KU764707	N/A
*K. tectonae*	MFLUCC 13-0470	KU144924	N/A	N/A
*K. thailandica*	MFLUCC 20-0116*	NR_178154	NG_088170	NG_087878
*K. thailandica*	MFLUCC 22-0020	ON878074	ON870387	ON870912
*K. thailandica*	MFLU 20-0263	MT985633	MT984443	MT984280
*K. thujina*	JF13210	KM982716	KM982718	KM982717
*K. tumidula*	CGMCC3.23629*	OQ645272	OQ645286	OQ645279
*K. vinigena*	CBS H-23378*	N/A	NG_075229	N/A
*K. weiningensis*	GZCC 24-0072*	PQ404851	PQ404855	PQ404858
*K. xishuangbannaensis*	ZHKUCC 22-0221	OP289563	OP303182	OP289565
*K. xishuangbannaensis*	ZHKUCC 22-0220*	OP289566	OP303181	OP289564
*K. xishuangbannaensis*	MFLUCC 23-0273*	OR762770	OR762778	OR764781
*K. xishuangbannaensis*	MFLUCC 23-0274	OR762769	OR762777	OR764780
*K. xishuiensis*	GZCC 24-0052*	PQ404850	PQ404854	PQ404857
*K. yadongensis*	HKAS 144543*	PQ684985	PV483694	PV483715
*K. yadongensis*	HKAS 144544	PQ684986	PV483695	PV483716
*K. yantingensis*	SICAUCC 23-0043*	PP060659	PP057952	PP003820
*K. yantingensis*	SICAUCC 23-0044	PP060660	PP057953	PP003821
*K. zizyphifolii*	MFLUCC 23-0270*	OR762768	OR762776	OR764779

Notes: New species established in this study are shown in bold. Ex-type strains are indicated using “*.” N/A: not available.

### Phylogenetic analyses

Nucleotide sequences of *Distoseptispora* and *Kirschsteiniothelia* were obtained from the National Center for Biotechnology Information (NCBI) database (https://www.ncbi.nlm.nih.gov/, accessed on 5 March 2026), and their corresponding GenBank accession numbers were extracted from the most recent version of the relevant research paper (Liu et al. 2025). MAFFT 7 (http://mafft.cbrc.jp/alignment/server/, accessed on 2 March 2026) was employed to conduct multiple sequence alignment for the nucleotide sequences of the three new taxa and the published reference sequences. Phylogenetic analyses based on maximum likelihood (ML) and Bayesian inference (BI) algorithms were carried out independently after registration on the CIPRES website (Miller et al. 2012). For ML phylogenetic inference, RAxML-HPC2 v.8.2.12 was run on XSEDE resources with the GTR+I+G nucleotide substitution model, and 1,000 rapid bootstrap replicates were performed to evaluate the confidence level (Stamatakis 2014). MrModeltest v.2.3 (Nylander 2004) was used to screen for the optimal evolutionary model, and BI analysis was performed using MrBayes 3.2.7a (XSEDE). FigTree v.1.4.3 (http://tree.bio.ed.ac.uk/software/figtree/, accessed on 2 March 2026) was used to visualize the inferred phylogenetic trees and reroot them with outgroups. The final versions of the phylogenetic trees were produced with Adobe Illustrator CC 2019, and the names of the new species were highlighted in red to achieve clear visual differentiation.

## Results

### Phylogenetic analyses

During the collection of dead wood samples and the isolation and identification of fungi in southern China, two fungal genera, *Distoseptispora* and *Kirschsteiniothelia*, exhibited high isolation frequencies and accounted for a significant proportion of the total isolated strains. Therefore, three new species belonging to the genera *Distoseptispora* and *Kirschsteiniothelia* are described in this paper.

#### Distoseptispora

**Figure 1.**
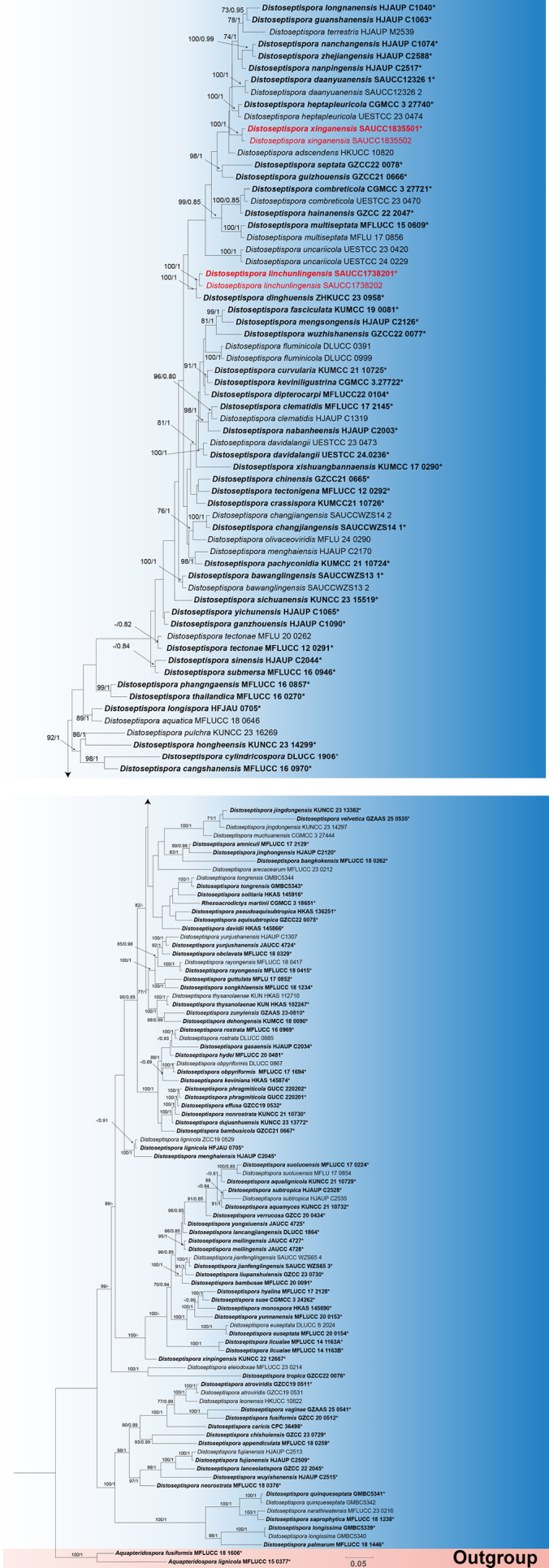
Maximum likelihood inference tree based on a combined dataset of analyzed ITS, LSU, *RPB2*, and *TEF1* sequences. The Bayesian inference posterior probability (right, BIPP ≥ 0.80) and maximum likelihood bootstrap value (left, MLBV ≥ 70%) are shown as MLBV/BIPP above the nodes. Those marked “*” in the tree represent ex-type or ex-epitype strains. Strains isolated in this study are indicated in red. The scale bar at the bottom indicates 0.1 substitutions per site.

Through phylogenetic analyses, the molecular dataset in this study comprised a total of 154 strains, including 119 *Distoseptispora* species. The closely related sister species *Aquapteridospora fusiformis* and *A. lignicola* (order *Distoseptisporales*) were selected as the outgroup, and the dataset contained 4,701 character sites, including ITS rDNA (1–746), LSU rDNA (747–2,567), *RPB2* (2,568–3,715), and *TEF1* (3,716–4,701). Among these, 2,425 sites were constant, 476 variable characters were parsimony-uninformative, and 1,800 were parsimony-informative. The GTR+I+G model was selected for ITS, *RPB2*, and *TEF1*, whereas the SYM+I+G model was applied to LSU. Given that the maximum likelihood (ML) tree and Bayesian inference (BI) tree were highly congruent in topology, the ML tree was selected as the representative topology for visualization (Fig. 1). Notably, the four *Distoseptispora* strains described in this study formed two independent clades in the phylogenetic tree, both with 100% bootstrap support and 1.00 posterior probability. Within these clades, *Distoseptispora linchunlingensis* sp. nov. was closely related to *D. dinghuensis*, whereas *Distoseptispora xinganensis* sp. nov. showed close phylogenetic affinity with *D. adscendens* and *D. heptapleuricola* (Fig. 1).

#### Kirschsteiniothelia

**Figure 2.**
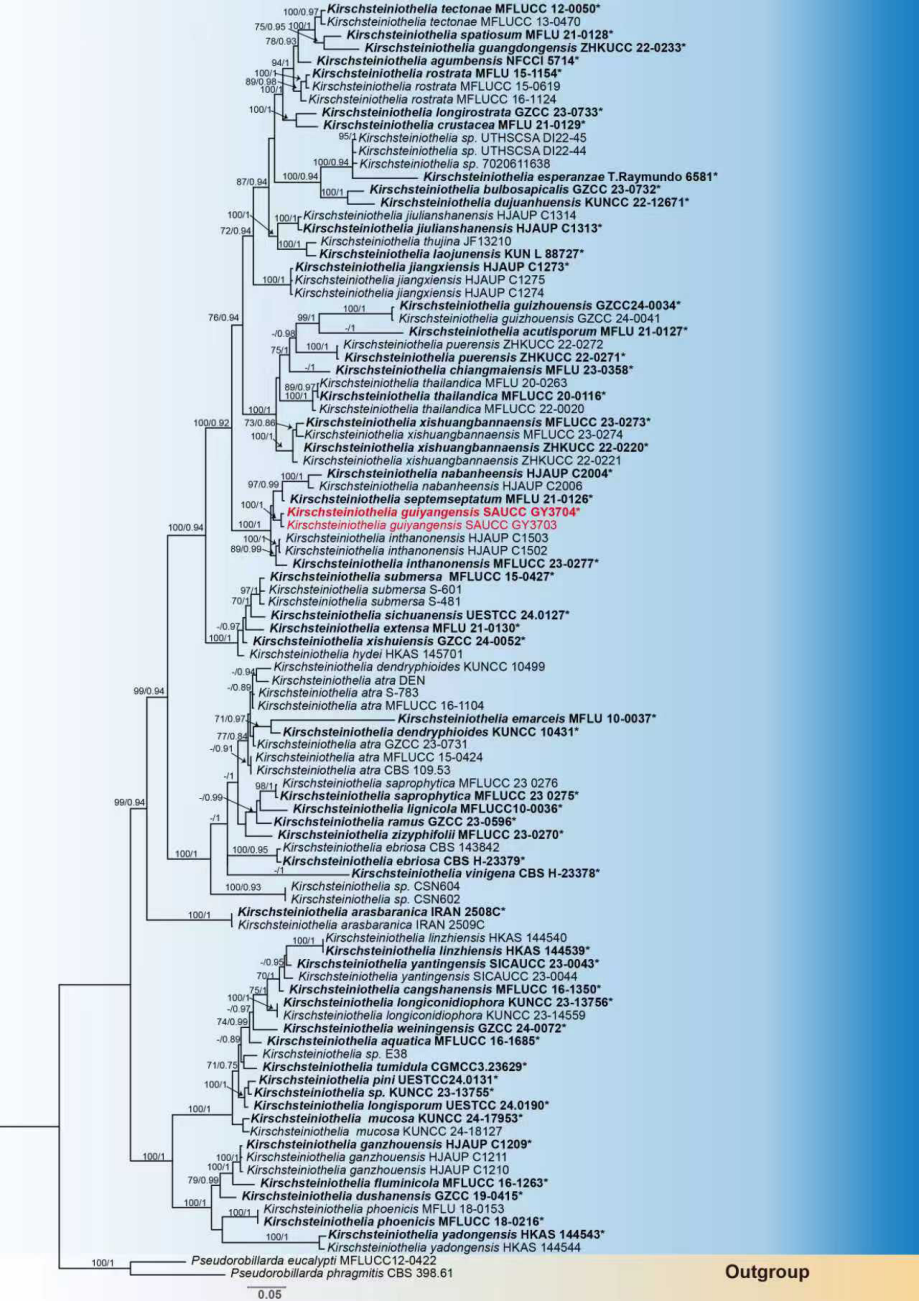
Maximum likelihood inference tree based on a combined dataset of analyzed ITS, LSU, and SSU sequences. The Bayesian inference posterior probability (right, BIPP ≥ 0.80) and maximum likelihood bootstrap value (left, MLBV ≥ 70%) are shown as MLBV/BIPP above the nodes. Those marked “*” in the tree represent ex-type or ex-epitype strains. Strains isolated in this study are indicated in red. The scale bar at the bottom indicates 0.1 substitutions per site.

Through phylogenetic analysis, the molecular dataset in this study comprised 99 strains, representing 57 *Kirschsteiniothelia* species and the outgroup taxa *Pseudorobillarda eucalypti* and *P. phragmitis*, and contained 2,758 character sites, including ITS rDNA (1–805), LSU rDNA (806–1,795), and SSU rDNA (1,796–2,758). Among these, 1,538 sites were constant, 297 variable characters were parsimony-uninformative, and 923 were parsimony-informative. The GTR+I+G model was selected for ITS and LSU, whereas the SYM+I+G model was applied to SSU. Given that the maximum likelihood (ML) tree and Bayesian inference (BI) tree were highly congruent in topology, the ML tree was selected as the representative topology for visualization (Fig. 2). The two *Kirschsteiniothelia* strains isolated in this study formed a single, fully supported clade in the phylogenetic tree, with bootstrap support of 100% and posterior probability of 1.00. Within this clade, the newly described species *Kirschsteiniothelia guiyangensis* sp. nov. exhibited a close phylogenetic relationship with *K. inthanonensis* and *K. septemseptatum* (Fig. 2).

### Taxonomy

#### Distoseptispora linchunlingensis

Taxon classificationFungiDistoseptisporalesDistoseptisporaceae

X.S. Wang, Y.Z. He, W.W. Liu, S. Wang sp. nov.

734A28CC-6F8A-5687-B93C-CD0476A2BBD8

Fungal Names: FN 573598

Fig. 3

##### Holotype.

China • Hainan Province, Sanya City, Jiyang District, Linchunling Forest Park, 18°15'46.02"N, 109°31'6.74"E, from decaying wood, 20 March 2025, W.W. Liu, holotype HMAS 354383, ex-type culture SAUCC1738201 (identical strain, alternative accession number SAUCC1738202).

**Figure 3.**
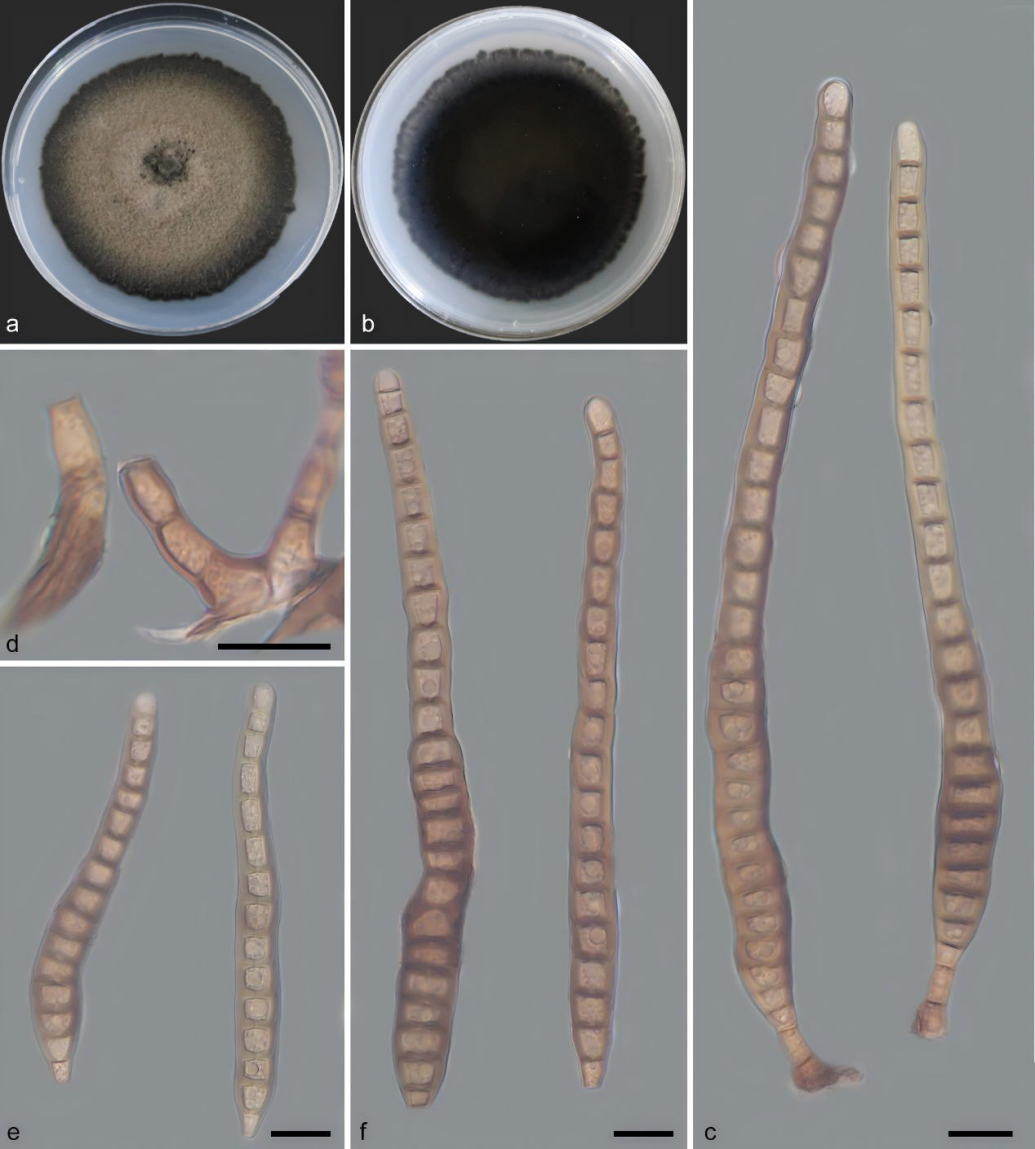
*Distoseptispora linchunlingensis* (holotype: HMAS 354383). **a, b**. Colony front and back after 21 days of culture on PDA; **c**. Conidiophores, conidiogenous cells, and conidia; **d**. Conidiophores; **e, f**. Conidia. Scale bars: 10 μm (**c–f**).

##### Etymology.

The specific epithet “*linchunlingensis”* indicates the location where the strains were collected, Linchunling Forest Park.

##### Description.

Saprobic on decaying wood. Sexual morph: undetermined. Asexual morph: Hyphomycetes. Mycelium pale brown with black margin, septate, branched, smooth, partly immersed in the substrate and partly aerial, with aerial mycelium gradually reduced in age; conidiophores cylindrical or elongated, filiform, myceliiform, branched, pale brown, 7.2–23.0 × 2.9–6.4 μm (x̄ = 14.1 × 4.0 μm, *n* = 10); conidiogenous cells terminal, inflated, cylindrical, smooth, pale brown, 31.1–42.8 × 8.4–9.3 μm (x̄ = 37.0 × 10.2 μm, *n* = 20); mature conidia solitary, apical, varying from cylindrical to slightly curved or obclavate, brown to pale brown, distoseptate, with smooth or finely verruculose walls; basal cell inflated and truncate, apical cell tapering and usually paler, 67.7–120.6 × 4.1–8.6 μm (x̄ = 93.8 × 6.1 μm, *n* = 20).

##### Culture characteristics.

Colonies on PDA attaining 67.8–69.2 mm diam. after 21 days of incubation at 25 °C in darkness, with a growth rate of 4.8–4.9 mm per day; colonies circular to subcircular, with deep black, irregularly dentate to fimbriate margins that contrast distinctly with the central area. Central surface pale brown to grayish brown, velvety, producing pale gray exudates; margins deep black to carbonaceous black, compact, velvety to short-velvety; reverse dark brown to black.

##### Notes.

Phylogenetic analyses showed that *Distoseptispora linchunlingensis* is closely related to *D. dinghuensis*. The nucleotide differences between *D. linchunlingensis* and *D. dinghuensis* are 22/575, 2/909, 7/953 and 0/911 in the ITS, LSU, *RPB2* and *TEF1* regions, respectively. Morphologically, *D. linchunlingensis* has shorter conidiophores (7.2–23.0 × 2.9–6.4 μm, x̄ = 14.1 × 4.0 μm, *n* = 10) and conidia (67.7–120.6 × 4.1–8.6 μm, x̄ = 93.8 × 6.1 μm, *n* = 20) than *D. dinghuensis* (conidiophores 22–48 × 3–6 μm, x̄ = 30.9 × 4.8 μm, *n* = 10; conidia 74–255 × 10–21 μm, x̄ = 150 × 13.8 μm, *n* = 30) (Dong et al. 2025). Therefore, based on phylogenetic and morphological evidence, *D. linchunlingensis* is introduced as a new species in the genus *Distoseptispora*.

#### Distoseptispora xinganensis

Taxon classificationFungiDistoseptisporalesDistoseptisporaceae

X.S. Wang, Y.Z. He, W.W. Liu, S. Wang sp. nov.

2E362CD4-B8BE-5C8E-84B5-C1708B6B6970

Fungal Names: FN 573599

Fig. 4

##### Holotype.

China • Guangxi Province, Guilin City, Xing’an County, Huajiang Yao Ethnic Township, 25°06'36"N, 109°13'48"E, from decaying wood, 20 May 2025, W.W. Liu, holotype HMAS 354412, ex-type culture SAUCC1835501 (identical strain, alternative accession number SAUCC1835502).

**Figure 4.**
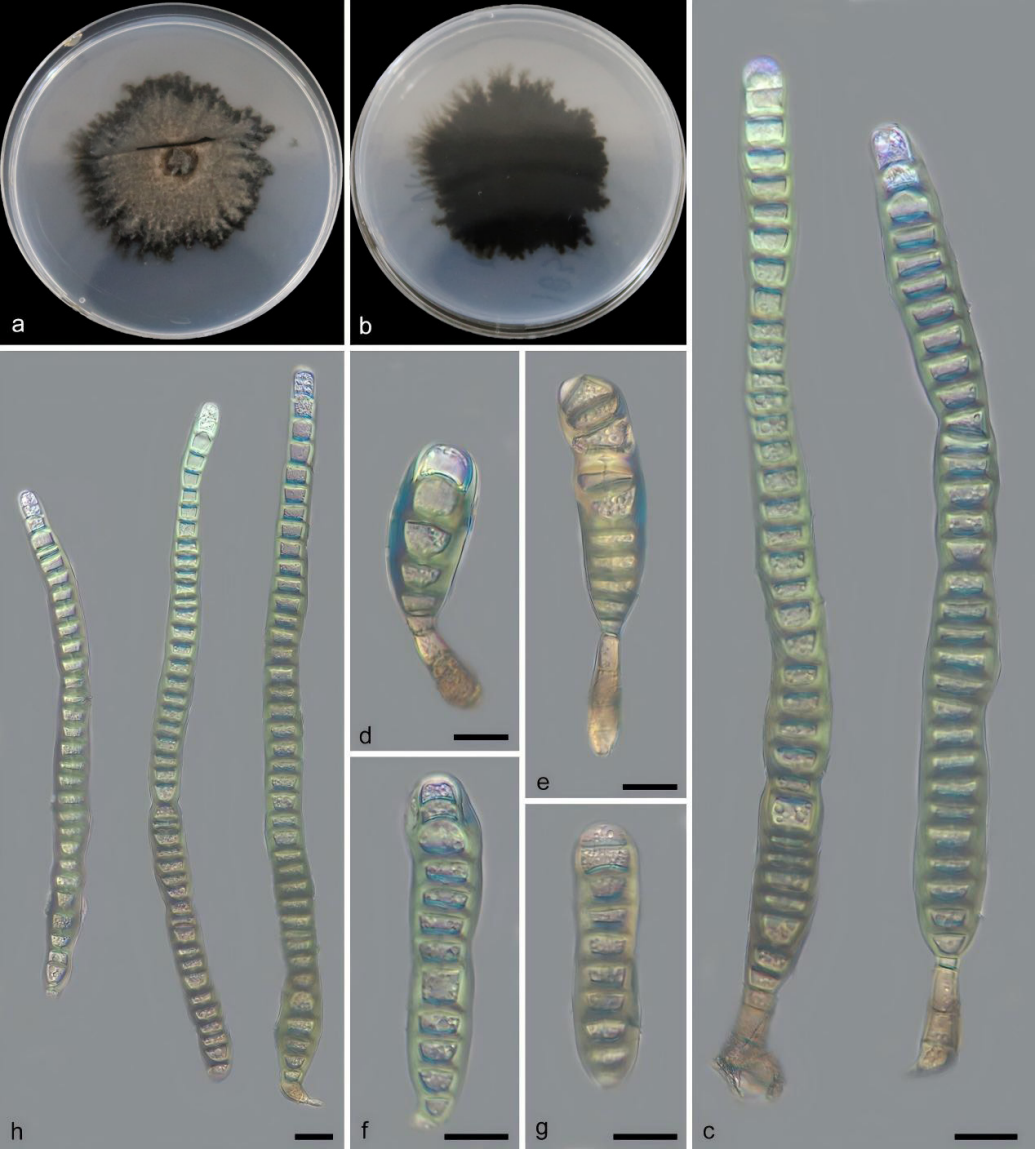
*Distoseptispora xinganensis* (holotype: HMAS 354412). **a, b**. Colony front and back after 21 days of culture on PDA; **c–e**. Conidiophores, conidiogenous cells, and conidia; **f–h**. Conidia. Scale bars: 10 μm (**c–h**).

##### Etymology.

The specific epithet “xinganensis” indicates the location where the strains were collected, Xing’an County.

##### Description.

Saprobic on decaying branches. Sexual morph: undetermined. Asexual morph: Hyphomycetes. Mycelium partly immersed in the substrate, internal hyphae pale brown, mostly dark olivaceous green externally; conidiophores macronematous, mononematous, solitary or in small fascicles, straight or slightly flexuous, unbranched, multi-septate, smooth-walled, olivaceous to brown, paler towards the apex, robust at the base, 16.0–19.4 × 4.4–10.3 μm (x̄ = 18.1 × 6.4 μm, *n* = 10). Conidiogenous cells terminal, monoblastic to polyblastic, cylindrical, hyaline to pale brown, smooth. Conidia terminal, solitary, cylindrical to obclavate, straight or slightly curved, apex rounded and hyaline, multi-septate, olivaceous to brown, with blue, yellow, and green tones, smooth-walled, length and septum number increasing with maturity, basal cell often slightly darker and thicker-walled than subsequent cells, 40.4–188.5 × 6.5–12.6 μm (x̄ = 118.4 × 9.4 μm, *n* = 20).

##### Culture characteristics.

Colonies on PDA reaching 50.2–57.6 mm diam. after 21 days at 25 °C in darkness, with a growth rate of 2.3–2.7 mm per day; colonies circular from above, central region pale grayish brown to grayish brown, dense and velvety, margin distinctly darker, deep black to ink black, floccose to villose, margin irregular, spreading radially or flabellately; reverse uniformly deep black to ink black; aerial mycelium moderately developed, rather low and dense in the central part, becoming gradually fluffy and higher toward the margin.

##### Notes.

Phylogenetic analyses based on ITS, LSU, *RPB2*, and *TEF1* sequences revealed that *Distoseptispora xinganensis* and *D. daanyuanensis*, *D. heptapleuricola*, *D. nanpingensis*, and *D. zhejiangensis* are located in the same clade with high support values in the phylogenetic tree and are closely related to each other. The nucleotide differences between *D. xinganensis* and *D. daanyuanensis* are 11/572 in ITS, 15/845 in LSU and 14/892 in *TEF1*; the nucleotide differences between *D. xinganensis* and *D. heptapleuricola* are 11/537 in ITS, 0/823 in LSU, 6/917 in *RPB2* and 21/924 in *TEF1*; the nucleotide differences between *D. xinganensis* and *D. nanpingensis* are 8/575 in ITS, 18/597 in LSU and 16/1059 in *RPB2*; and the nucleotide differences between *D. xinganensis* and *D. zhejiangensis* are 12/577 in ITS, 24/604 in LSU and 20/983 in *TEF1*, respectively. From a morphological perspective, *Distoseptispora xinganensis* differs from *D. daanyuanensis*, *D. heptapleuricola*, *D. nanpingensis*, and *D. zhejiangensis* in conidiophores, conidiogenous cells, and conidia. The conidiophores of *D. xinganensis* are 16.0–19.4 × 4.4–10.3 μm (x̄ = 18.1 × 6.4 μm, *n* = 10), which are shorter than those of *D. daanyuanensis* (25.5–46.7 × 1.3–2.4 μm, x̄ = 38.4 × 1.8 μm, *n* = 20), *D. heptapleuricola* (7–29 × 6–11 μm, x̄ = 17 × 7.5 μm, *n* = 20), *D. nanpingensis* (8.5–28 × 5–7 μm, x̄ = 18.1 × 6.1 μm, *n* = 10), and *D. zhejiangensis* (16–56 × 13.5–20 μm, x̄ = 38 × 9.7 μm, *n* = 15). Additionally, the conidia of *D. xinganensis* measure 40.4–188.5 × 6.5–12.6 μm (x̄ = 118.4 × 9.4 μm, *n* = 20), which are also smaller than those of *D. daanyuanensis* (23.1–696.2 × 9.3–17.9 μm, x̄ = 302.4 × 11.3 μm, *n* = 25), *D. heptapleuricola* ((67–)115–500(–590) × 10–19 μm, x̄ = 250 × 13 μm, *n* = 30) (Zhang et al. 2022), *D. nanpingensis* (169–282 × 12–17.5 μm, x̄ = 227.1 × 14.8 μm, *n* = 20) and *D. zhejiangensis* (128.1–261.7 × 18.7–26.7 μm, x̄ = 161.1 × 23.5 μm, *n* = 30). Combined with phylogenetic and morphological evidence, *D. xinganensis* is consequently confirmed as a new species of the genus *Distoseptispora*.

#### Kirschsteiniothelia guiyangensis

Taxon classificationFungiKirschsteiniothelialesCorylaceae

X.S. Wang, Y.Z. He, W.W. Liu, S. Wang sp. nov.

DFCCD242-FA73-5C53-90FD-73C64C7090B8

Fungal Names: FN 573600

Fig. 5

##### Holotype.

China • Guizhou Province, Guiyang City, Huaxi District, Mengguan Miao and Bouyei Ethnic Township, Mengxi Road, 18°15'46"N, 109°31'07"E, from decaying wood, 23 September 2023, W.W. Liu, holotype HMAS 354384, ex-type culture SAUCC GY3703 (identical strain, alternative accession number SAUCC GY3704).

**Figure 5.**
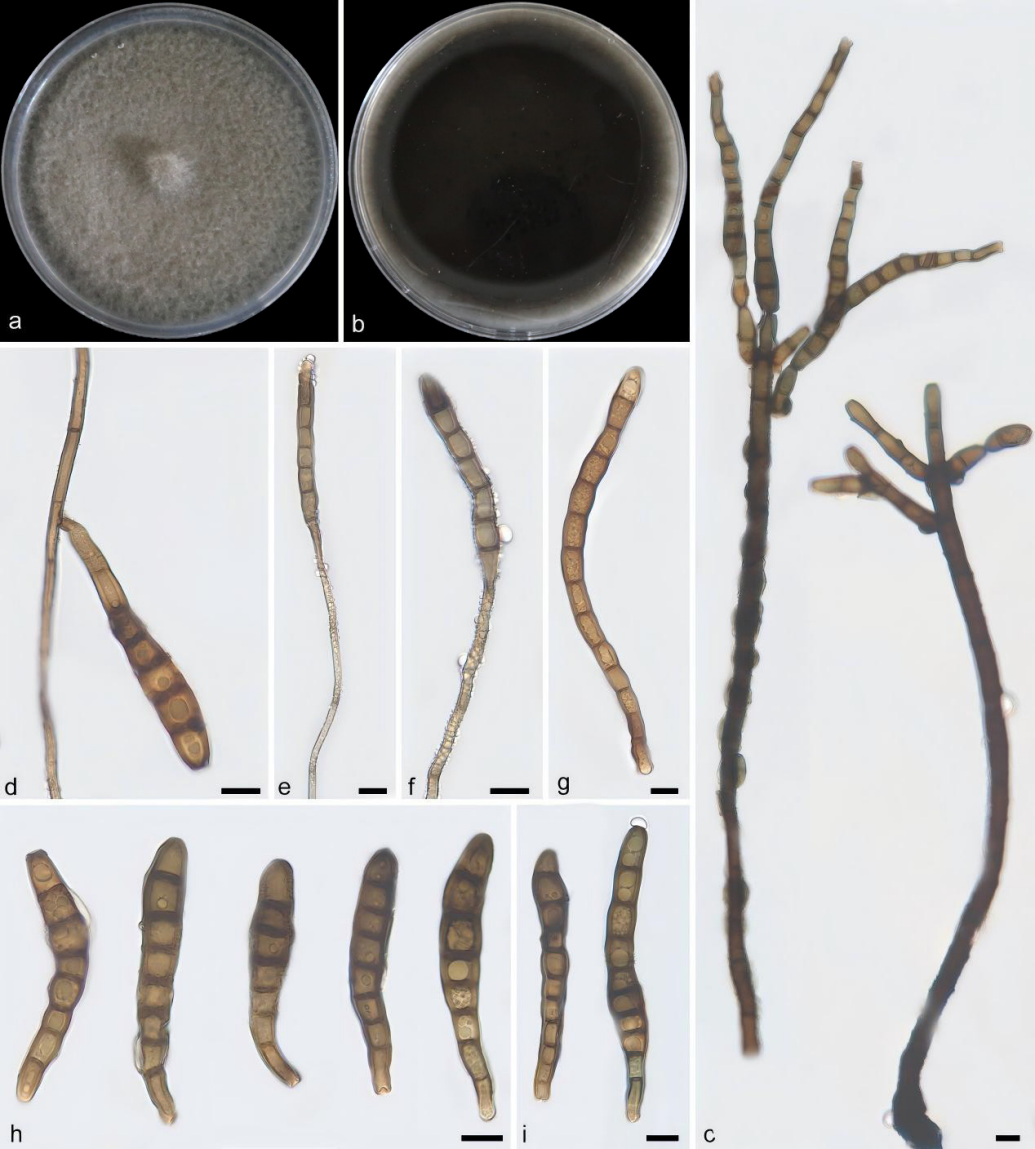
*Kirschsteiniothelia guiyangensis* (holotype: HMAS 354384). **a, b**. Colony front and back after 21 days of culture on PDA; **c–f**. Conidiophores, conidiogenous cells, and conidia; **g–i**. Conidia. Scale bars: 10 μm (**c–i**).

##### Etymology.

The specific epithet “guiyangensis” indicates the location where the strains were collected, Guiyang City.

##### Description.

Saprobic on decaying wood. Sexual morph: undetermined. Asexual morph: Hyphomycetes. Mycelium partly immersed in the substrate, internal hyphae grey; conidiophores solitary, straight or slightly curved, filiform, multi-septate, brown to dark brown, 29.8–301.8 × 3.6–9.5 μm (x̄ = 174.7 × 8.3 μm, *n* = 10); conidiogenous cells integrated, terminal, polyblastic, subcylindrical to ampulliform, light brown to brown, smooth, with several distinct conidiogenous loci; conidia solitary, dry, apical, obclavate to fusiform, straight or slightly curved, brown to dark brown, euseptate with 3–8 transverse septa, smooth-walled, apex subacute to obtuse, base truncate, 55.2–90.1 × 5.4–11.6 μm (x̄ = 67.3 × 9.5 μm, *n* = 20).

##### Culture characteristics.

Colonies on PDA reaching 80.3–84.5 mm diam. after 21 days at 25 °C in darkness, with a growth rate of 3.8–4.0 mm per day; colonies fast-growing, circular, spreading radially, with a well-defined and intact margin, central area slightly raised; texture velvety to cottony, aerial mycelium abundant and dense, growing radially from the center outward, white to pale gray; reverse dark brown to black, darker in the central region.

##### Notes.

Phylogenetic analyses showed that *Kirschsteiniothelia guiyangensis* is closely related to *K. inthanonensis*, *K. septemseptatum*, and *K. nabanheensis*. The nucleotide differences between *K. guiyangensis* and *K. inthanonensis* are 23/552 in ITS, 13/576 in LSU and 4/951 in SSU; the nucleotide differences between *K. guiyangensis* and *K. septemseptatum* are 21/504 in ITS, 8/852 in LSU and 9/748 in SSU; and the nucleotide differences between *K. guiyangensis* and *K. nabanheensis* are 29/552 in ITS, 28/596 in LSU and 38/855 in SSU, respectively. Morphologically, *K. guiyangensis* has much shorter and smaller conidiophores (29.8–301.8 × 3.6–9.5 μm, x̄ = 174.7 × 8.3 μm, *n* = 10) and conidia (55.2–90.1 × 5.4–11.6 μm, x̄ = 67.3 × 9.5 μm, *n* = 20) than *K. inthanonensis* (conidiophores 611–1549 × 2.5–6.6 μm, x̄ = 1070 × 4.1 μm, *n* = 20; conidia 24–230 × 5.7–14.3 μm, x̄ = 101 × 9 μm, *n* = 15) (de Farias et al. 2024). The conidiophores of *K. guiyangensis* (29.8–301.8 × 3.6–9.5 μm, x̄ = 174.7 × 8.3 μm, *n* = 10) are smaller than those of *K. septemseptatum* (250–580 μm, x̄ = 415 μm, *n* = 20) and *K. nabanheensis* (320–588 × 8–12 μm, x̄ = 405 × 9.5 μm, *n* = 15), whereas the conidia of *K. guiyangensis* (55.2–90.1 × 5.4–11.6 μm, x̄ = 67.3 × 9.5 μm, *n* = 20) are larger than those of *K. septemseptatum* (25–55 μm, x̄ = 41 μm, *n* = 20) but smaller than those of *K. nabanheensis* (32–112 × 8–12 μm, x̄ = 55.5 × 10 μm, *n* = 25). Combined morphological distinctiveness and consistent molecular divergence clearly rule out the possibility of intraspecific phenotypic variation, and the stable phenotypic differences, together with significant genetic divergence in the ITS, LSU, and SSU loci, provide sufficient evidence to establish the present isolate as a novel species of *Kirschsteiniothelia*, rather than a morphological variant of a known taxon.

## Discussion

In this study, three new fungal species, *Distoseptispora linchunlingensis*, *D. xinganensis* and *Kirschsteiniothelia guiyangensis*, were discovered and described from decaying forest plant debris in Hainan and Guizhou provinces, China. These findings further confirm that substantial undiscovered fungal diversity still exists in forest ecosystems of tropical and subtropical regions in China (Hyde et al. 2020). Although the global number of fungal species is extremely high, only a small fraction has been formally described (Hawksworth and Lücking 2017). Saprotrophic groups, such as dematiaceous hyphomycetes, are particularly understudied, largely due to difficulties in isolation and cultivation, as well as insufficient survey coverage, leading to severe underestimation of their species richness (Seifert et al. 2011). The new taxa obtained in this study further demonstrate that traditional investigation methods are insufficient to uncover the true diversity of saprotrophic fungi, and the integrative approach combining morphology and multi-locus phylogenetic analysis remains a reliable strategy for the discovery and delimitation of new species (Manawasinghe et al. 2021; Shenoy 2025).

Two novel species of *Distoseptispora*, namely *D. linchunlingensis* and *D. xinganensis*, proposed in this study greatly expand the morphological variation range, geographical distribution, and substrate adaptability of the genus. Morphologically, these two taxa display unique characteristics in conidiophores, conidiogenous cells, and conidia. Among them, *D. linchunlingensis* is closely related to *D. dinghuensis* and morphologically possesses shorter conidiophores and conidia than the latter, whereas *D. xinganensis* resides in the same sister clade as *D. daanyuanensis*, *D. heptapleuricola*, *D. nanpingensis*, and *D. zhejiangensis* and also has shorter conidiophores and conidia than the four aforementioned species. These characteristics not only supplement and enrich the phenotypic morphological database of *Distoseptispora* but also distinguish the two new taxa from all previously recorded congeners. Numerous new *Distoseptispora* taxa have been discovered in tropical and subtropical regions since the establishment of the genus (Su et al. 2016), whereas most sampling surveys have been limited to narrow substrate types, such as decaying wood, resulting in unbalanced regional research (Zhai et al. 2022). In addition, the discovery of the genus in Hainan further confirms its distribution records in tropical humid forests of southern China and supports the viewpoint that *Distoseptispora* exhibits high species richness in South China (Hu et al. 2023). Taxonomically, *Distoseptispora* shares highly similar morphological features with its allied genera, which leads to ambiguity in morphology-based species delimitation (Liu et al. 2023). Based on the combined analyses of ITS, LSU, *RPB2*, and *TEF1* molecular markers, this study successfully resolved the interspecific phylogenetic relationships and clearly differentiated the two new species from their closely related taxa. This multi-locus dataset provides a stable molecular reference for subsequent species delimitation of *Distoseptispora* and confirms the necessity of integrating molecular phylogenetic analysis with morphological characteristics for accurate species identification of this genus. *Kirschsteiniothelia* has recently undergone considerable species enrichment, and this study describes *Kirschsteiniothelia guiyangensis* sp. nov. to improve the taxonomic integrity of this genus. Morphologically, the new species exhibits clear morphological divergence from known congeners, broadening the morphological variation range of terrestrial lignicolous *Kirschsteiniothelia*. Ecogeographically, this fungus was collected from decaying wood in subtropical karst habitats of Guiyang, representing a new distribution record for the genus in karst ecosystems and improving current knowledge of its biogeographic pattern in southwest China. As a wood-associated saprobe without host specificity, *K. guiyangensis* supports the broad substrate adaptability of terrestrial members, whereas occasional freshwater and pathogenic congeners collectively reflect the wide ecological amplitude of this genus (Bao 2018; Nishi et al. 2018). Consistent with most published taxa, only the asexual morph was obtained in this new species. The insufficient documentation of sexual morphs remains a major constraint for accurate species delimitation in *Kirschsteiniothelia*, requiring further sampling and cultivation to supplement holomorph data (Chethana et al. 2021; Maharachchikumbura et al. 2021). Overall, *K. guiyangensis* provides reliable morphological, geographical, and ecological information to facilitate subsequent species boundary clarification and phylogenetic analysis of the genus. Overall, the three newly discovered fungal species have important taxonomic and ecological significance for *Distoseptispora* and *Kirschsteiniothelia*. These taxa not only expand the morphological diversity, geographical distribution, and substrate adaptability of the two genera but also provide reliable molecular and morphological evidence for optimizing the species delimitation criteria of dematiaceous hyphomycetes. Hainan tropical rainforests and Guizhou karst forests have been confirmed as vital biodiversity hotspots for saprobic fungi. Although saprobic fungi play an irreplaceable ecological role in forest litter decomposition, nutrient cycling and carbon pool maintenance, studies of their taxonomy and diversity are still relatively insufficient. In the future, continuous targeted surveys and sampling in subtropical and tropical forests of southern China, combined with phylogenetic and morphological analyses, will help discover more cryptic species, continuously improve the taxonomic system of dematiaceous hyphomycetes and related groups, and further clarify their species diversity, phylogenetic relationships, and ecological functions.

## Conclusion

After extensive sampling of decaying wood specimens from multiple regions in southern China, several new fungal species were isolated. Among them, two new species of *Distoseptispora* and one new species of *Kirschsteiniothelia* were delineated and confirmed based on molecular phylogenetic analyses combined with morphological observations. All fungal strains in this study were recovered from decaying wood and are regarded as saprobic fungi. Based on the present results, substrate-targeted sampling of decaying wood can facilitate the efficient exploration and systematic documentation of undiscovered novel species within these two fungal genera.

## Supplementary Material

XML Treatment for Distoseptispora linchunlingensis

XML Treatment for Distoseptispora xinganensis

XML Treatment for Kirschsteiniothelia guiyangensis

## Additional information

### Artificial Intelligence (AI) use

The authors accept full responsibility for the content of the manuscript, including the disclosure of any use of AI.

No AI tools were used in the preparation of this manuscript.

### Author ORCIDs

Y. He https://orcid.org/0009-0005-7851-7055

X. Wang https://orcid.org/0009-0005-1127-7368

X. Yan https://orcid.org/0009-0005-6257-3490

W. Liu https://orcid.org/0009-0009-3477-194X

Y. Jiang https://orcid.org/0009-0003-1292-610X

C. Ai https://orcid.org/0009-0002-3934-7944

J. Sheng https://orcid.org/0009-0003-3882-6748

X. Zhang https://orcid.org/0000-0001-9733-8494

S. Wang https://orcid.org/0000-0002-7376-7638

### Data availability

All of the data that support the findings of this study are available in the main text or Supplementary Information.
